# Cosine Similarity Measure between Hybrid Intuitionistic Fuzzy Sets and Its Application in Medical Diagnosis

**DOI:** 10.1155/2018/3146873

**Published:** 2018-10-17

**Authors:** Donghai Liu, Xiaohong Chen, Dan Peng

**Affiliations:** ^1^Department of Mathematics, Hunan University of Science and Technology, Xiangtan, China; ^2^Hunan University of Commerce, Changsha, China

## Abstract

In this paper, a cosine similarity measure between hybrid intuitionistic fuzzy sets is proposed. The aim of the paper is to investigate the cosine similarity measure with hybrid intuitionistic fuzzy information and apply it to medical diagnosis. Firstly, we construct the cosine similarity measure between hybrid intuitionistic fuzzy sets, and the relevant properties are also discussed. In order to obtain a reasonable evaluation in group decision, the weight of experts under different attributes is determined by the projection of individual decision information on the ideal decision information, where the ideal decision information is the average values of each expert's evaluation. Furthermore, we propose a decision method for medical diagnosis based on the cosine similarity measure between hybrid intuitionistic fuzzy sets, and the patient can be diagnosed with the disease according to the values of proposed cosine similarity measure. Finally, an example is given to illustrate feasibility and effectiveness of the proposed cosine similarity measure, which is also compared with the existing similarity measures.

## 1. Introduction

A similarity measure is an important tool for determining the degree of similarity between two objects in many fields, such as pattern recognition, medical diagnosis, and so on. Many similarity measures have been introduced [1–8]. Among them, some similarity measures of intuitionistic fuzzy sets (IFSs) have been proposed. For example, Li and Cheng [3] proposed a similarity measure between IFSs and applied it to pattern recognition. Huang and Yang [2] defined the similarity measure between IFSs based on the Hausdorff distance and used it to calculate the degree of similarity between IFSs. Nguuen [9] proposed a new knowledge-based similarity measure between IFSs and applied it to pattern recognition. However, due to the complexity and uncertainty of the decision-making environment, the membership degree and nonmembership degree of IFS need to be expressed by interval rather than the numerical value. Motivated by this, Atanassov and Gargov [10] introduced the concept of interval-valued intuitionistic fuzzy set (IVIFS), which is a generalization of IFS. Xu [11] proposed some distance and similarity measures between IVIFSs and applied them to pattern recognition.

On the other hand, the cosine similarity measure based on Bhattacharyya distance was first proposed in Bhattacharyya [12]. Ye [7] proposed a cosine similarity measure for IFSs (*C*_IFS_) and applied it to pattern recognition. Furthermore, Ye [13] proposed the cosine similarity measure for IVIFSs (*C*_IVIFS_) and applied it to group decision-making problems. However, in the complex group decision-making problem, it is difficult to use a single value to express the alternative under all attributes. Because some attributes might be represented by IFSs, but other attributes are suitable to be represented by IVIFSs. At this time, the people should use hybrid intuitionistic fuzzy set to make a decision. However, the existing methods can not deal with the hybrid fuzzy information. As far as we know, no people studied the cosine similarity measure between hybrid IFSs. Motivated by this, we will introduce the cosine similarity measure with hybrid intuitionistic fuzzy information (*C*_HIFS_) in this paper. This generalization makes the *C*_HIFS_ measure includes *C*_IFS_ measure and *C*_IVIFS_ measure as particular case.

In addition, applying the *C*_HIFS_ measure to group decision-making problems is very interesting. For example, Zhou and Wahab [14] use transmissibility incorporated with cosine similarity measure to investigate the structural damage detection. Furthermore, Zhou et al. [15] apply transmissibility function with distance measure to separate the intact patterns apart from the damaged pattern. In group decision-making problems, the weight of the experts under different attributes can be obtained by using the projection of individual decision information on the ideal decision information. Then, we aggregate all individual decisions into a collective one and apply the proposed cosine similarity measure between hybrid intuitionistic fuzzy sets to medical diagnosis.

The rest of the paper is organized as follows. In Section 2, we review the cosine similarity measure for IFSs and IVIFSs. In Section 3, we propose the *C*_HIFS_ measure, some properties are also analyzed. In Section 4, we propose a decision method for medical diagnosis based on the cosine similarity measure between hybrid intuitionistic fuzzy sets. In Section 5, an example is given to illustrate the feasibility and effectiveness of the proposed *C*_HIFS_ measure. Finally, the conclusion and further research are discussed in Section 6.

## 2. Preliminaries

Throughout this paper, let *X*={*x*_1_, *x*_2_, ..., *x*_*n*_} be a finite universal set. In this section, we briefly review the IFSs and IVIFSs, the cosine similarity measure between IFSs, and the cosine similarity measure between IVIFSs.

### 2.1. Intuitionistic Fuzzy Set

*Definition 1*. Let *X* be a fixed set, an intuitionistic fuzzy set (IFS) *A* in *X* is defined as:

(1) $$\begin{array}{c} A=\left\{\left(x_{j},\mu_{A}\left(x_{j}\right),\nu_{A}\left(x_{j}\right)\left|\;x_{j}\in X\right.\right)\right\}, \end{array}$$

where the functions *μ*_*A*_(*x*_*j*_) and *ν*_*A*_(*x*_*j*_) represent the membership degree and nonmembership degree of the element *x*_*j*_ to the set *A*, respectively, such that 0 ≤ *μ*_*A*_(*x*_*j*_)+*ν*_*A*_(*x*_*j*_) ≤ 1∀ *x*_*j*_ ∈ *X*.

**Figure 1:**
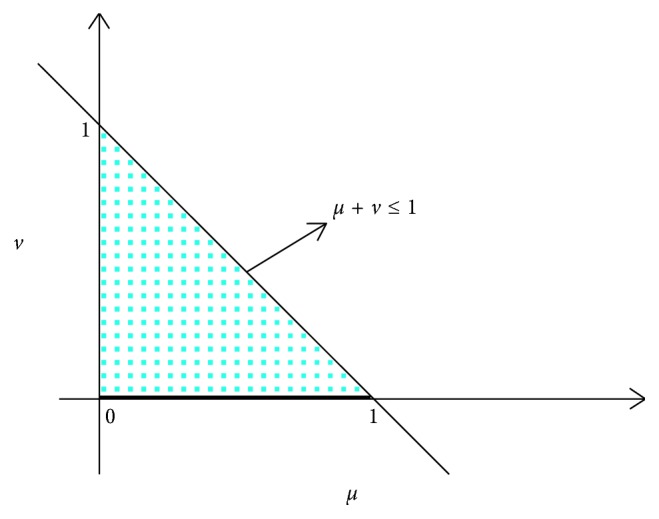
The membership degree of IFS.

The intuitionistic fuzzy index *π*_*A*_(*x*_*j*_)=1 − *μ*_*A*_(*x*_*j*_) − *ν*_*A*_(*x*_*j*_), and we have 0 ≤ *π*_*A*_(*x*_*j*_) ≤ 1. For example, *A*=(0.4, 0.3) is an intuitionistic fuzzy number, and *π*_*A*_=0.3. The space of membership degree of IFS is shown in Figure 1.

In particular, when *X* has only one element, the IFS *A*={(*x*_*j*_, *μ*_*A*_(*x*_*j*_), *ν*_*A*_(*x*_*j*_)|*x*_*j*_ ∈ *X*)} is reduced to *A*=(*μ*_*A*_(*x*_*j*_), *ν*_*A*_(*x*_*j*_)), which we call it an intuitionistic fuzzy number (IFN).

For any two IFSs *A*=(*x*_*j*_, *μ*_*A*_(*x*_*j*_), *ν*_*A*_(*x*_*j*_)) and *B*=(*x*_*j*_, *μ*_*B*_(*x*_*j*_), *ν*_*B*_(*x*_*j*_)), the following operations are true [16]:

1. *A*+*B*=(*μ*_*A*_(*x*_*j*_)+*μ*_*B*_(*x*_*j*_) − *μ*_*A*_(*x*_*j*_)*μ*_*B*_(*x*_*j*_), *ν*_*A*_(*x*_*j*_)*ν*_*B*_(*x*_*j*_))
2. *λA*=(1 − (1 − *μ*_*A*_(*x*_*j*_))^*λ*^, (*ν*_*A*_(*x*_*j*_))^*λ*^), *λ* > 0
3. *A*=*B* *if* *μ*_*A*_(*x*_*j*_)=*μ*_*B*_(*x*_*j*_), *ν*_*A*_(*x*_*j*_)=*ν*_*B*_(*x*_*j*_)

The results of the operations *A*+*B* and *λA* are still IFSs.

### 2.2. Interval-Valued Intuitionistic Fuzzy Set

*Definition 2*. Let *X* be a fixed set, an interval-valued intuitionistic fuzzy set $\tilde{A}$ is defined as follows:

(2) $$\begin{array}{c} \tilde{A}=\left\{\left(x_{j},\left[\mu_{\tilde{A}L}\left(x_{j}\right),\mu_{\tilde{A}U}\left(x_{j}\right)\right],\left[\nu_{\tilde{A}L}\left(x_{j}\right),\nu_{\tilde{A}U}\left(x_{j}\right)\right]\right)\left|\;x_{j}\in X\right.\right\}, \end{array}$$

where

(3) $$\begin{array}{c} \mu_{\tilde{A}L}\left(x_{j}\right)\geq 0,\nu_{\tilde{A}L}\left(x_{j}\right)\geq 0;0\leq \mu_{\tilde{A}U}\left(x_{j}\right)+\nu_{\tilde{A}U}\left(x_{j}\right)\leq 1,\;\forall x_{j}\in X. \end{array}$$

The interval-valued intuitionistic fuzzy index is defined as $\pi_{\tilde{A}}\left(x_{j}\right)=\left[\pi_{\tilde{A}L}\left(x_{j}\right),\pi_{\tilde{A}U}\left(x_{j}\right)\right]$, where

(4) $$\begin{array}{c} \pi_{\tilde{A}L}\left(x_{j}\right)=1-\mu_{\tilde{A}U}\left(x_{j}\right)-\nu_{\tilde{A}U}\left(x_{j}\right),\pi_{\tilde{A}U}\left(x_{j}\right)=1-\mu_{\tilde{A}L}\left(x_{j}\right)-\nu_{\tilde{A}L}\left(x_{j}\right). \end{array}$$

For example, $\tilde{A}=\left(\left[0.3,0.4\right],\left[0.1,0.4\right]\right)$ is an interval-valued intuitionistic fuzzy number, the fuzzy index $\pi_{\tilde{A}}\left(x_{j}\right)=\left[0.2,0.6\right]$.

***Remark 1*.**

If $\mu_{\tilde{A}L}\left(x_{j}\right)=\mu_{\tilde{A}U}\left(x_{j}\right),\nu_{\tilde{A}L}\left(x_{j}\right)=\nu_{\tilde{A}U}\left(x_{j}\right),$ then the interval-valued intuitionistic fuzzy set is reduced to intuitionistic fuzzy set.

When the set *X* has only one element, the IVIFS $\tilde{A}=\left\{\left(x_{j},\left[\mu_{\tilde{A}L}\left(x_{j}\right),\mu_{\tilde{A}U}\left(x_{j}\right)\right],\left[\nu_{\tilde{A}L}\left(x_{j}\right),\nu_{\tilde{A}U}\left(x_{j}\right)\right]\right)\left|x_{j}\in X\right.\right\}$ is reduced to $\tilde{A}=\left(\left[\mu_{\tilde{A}L}\left(x_{j}\right),\mu_{\tilde{A}U}\left(x_{j}\right)\right],\left[\nu_{\tilde{A}L}\left(x_{j}\right),\nu_{\tilde{A}U}\left(x_{j}\right)\right]\right)$, which we call it an interval-valued intuitionistic fuzzy number(IVIFN).

Let $\tilde{A}=\left(x_{j},\left[\mu_{\tilde{A}L}\left(x_{j}\right),\mu_{\tilde{A}U}\left(x_{j}\right)\right],\left[\nu_{\tilde{A}L}\left(x_{j}\right),\nu_{\tilde{A}U}\left(x_{j}\right)\right]\right)$ and $\tilde{B}=\left(x_{j},\left[\mu_{\tilde{B}L}\left(x_{j}\right),\mu_{\tilde{B}U}\left(x_{j}\right)\right],\left[\nu_{\tilde{B}L}\left(x_{j}\right),\nu_{\tilde{B}U}\left(x_{j}\right)\right]\right)$ be two IVIFSs, the following operations are true [17]:

1. $\tilde{A}+\tilde{B}=\left(\left[\mu_{\tilde{A}L}\left(x_{j}\right)+\mu_{\tilde{B}L}\left(x_{j}\right)-\mu_{\tilde{A}L}\left(x_{j}\right)\mu_{\tilde{B}L}\left(x_{j}\right),\mu_{\tilde{A}U}\left(x_{j}\right)+\mu_{\tilde{B}U}\left(x_{j}\right)-\mu_{\tilde{A}U}\left(x_{j}\right)\mu_{\tilde{B}U}\left(x_{j}\right)\right]\right.$, $\left(\left[\nu_{\tilde{A}L}\left(x_{j}\right)\nu_{\tilde{B}L}\left(x_{j}\right),\nu_{\tilde{A}U}\left(x_{j}\right)\nu_{\tilde{B}U}\left(x_{j}\right)\right]\right)$
2. $\lambda \tilde{A}=\left(\left[1-{\left(1-\mu_{\tilde{A}L}\left(x_{j}\right)\right)}^{\lambda },1-{\left(1-\mu_{\tilde{A}U}\left(x_{j}\right)\right)}^{\lambda }\right],\left[{\left(\nu_{\tilde{A}L}\left(x_{j}\right)\right)}^{\lambda },{\left(\nu_{\tilde{A}U}\left(x_{j}\right)\right)}^{\lambda }\right]\right),\lambda >0$
3. $\tilde{A}=\tilde{B}$ if $\mu_{\tilde{A}L}\left(x_{j}\right)=\mu_{\tilde{B}L}\left(x_{j}\right),\mu_{\tilde{A}U}\left(x_{j}\right)=\mu_{\tilde{B}U}\left(x_{j}\right),\nu_{\tilde{A}L}\left(x_{j}\right)=\nu_{\tilde{B}L}\left(x_{j}\right)$ and $\nu_{\tilde{A}U}\left(x_{j}\right)=\nu_{\tilde{B}U}\left(x_{j}\right)$

### 2.3. Cosine Similarity Measures for IFSs or IVIFSs

*Definition 3* (Ye [7]). Let *A*=(*μ*_*A*_(*x*_*j*_), *ν*_*A*_(*x*_*j*_)) and *B*=(*μ*_*B*_(*x*_*j*_), *ν*_*B*_(*x*_*j*_)) be two IFSs in *X*, the cosine similarity measure between *A* and *B* is defined as follows:

(5) $$\begin{array}{c} C_{\text{IFS}}\left(A,B\right)=\frac{1}{n}\overset{n}{\underset{i=1}{\sum }}\frac{\mu_{A}\left(x_{i}\right)\mu_{B}\left(x_{i}\right)+\nu_{A}\left(x_{i}\right)\nu_{B}\left(x_{i}\right)}{\sqrt{\mu_{A}^{2}\left(x_{i}\right)+\nu_{A}^{2}\left(x_{i}\right)}\sqrt{\mu_{B}^{2}\left(x_{i}\right)+\nu_{B}^{2}\left(x_{i}\right)}}. \end{array}$$

The cosine similarity measure between two IFSs *A* and *B* satisfies the following properties:

1. 0 ≤ *C*_IFS_(*A*, *B*) ≤ 1
2. *C*_IFS_(*A*, *B*)=*C*_IFS_(*B*, *A*)
3. *C*_IFS_(*A*, *B*)=1 if *A*=*B*

*Definition 4* (Ye [13]). Let $\tilde{A}=\left(\left[\mu_{\tilde{A}L}\left(x_{j}\right),\mu_{\tilde{A}U}\left(x_{j}\right)\right],\left[\nu_{\tilde{A}L}\left(x_{j}\right),\nu_{\tilde{A}U}\left(x_{j}\right)\right]\right)$ and $\tilde{B}=\left(\left[\mu_{\tilde{B}L}\left(x_{j}\right),\mu_{\tilde{B}U}\left(x_{j}\right)\right]\right)$, $\left[\nu_{\tilde{B}L}\left(x_{j}\right),\nu_{\tilde{B}U}\left(x_{j}\right)\right]$ be two IVIFSs in *X*, the cosine similarity measure between two IVIFSs $\tilde{A}$ and $\tilde{B}$ is defined as follows:

(6) $$\begin{array}{c} C_{\text{IVIFS}}\left(\tilde{A},\tilde{B}\right)=\frac{1}{n}\overset{n}{\underset{i=1}{\sum }}\frac{\mu_{\tilde{A}L}\left(x_{i}\right)\mu_{\tilde{B}L}\left(x_{i}\right)+\mu_{\tilde{A}U}\left(x_{i}\right)\mu_{\tilde{B}U}\left(x_{i}\right)+\nu_{\tilde{A}L}\left(x_{i}\right)\nu_{\tilde{B}L}\left(x_{i}\right)+\nu_{\tilde{A}U}\left(x_{i}\right)\nu_{\tilde{B}U}\left(x_{i}\right)+\pi_{\tilde{A}L}\left(x_{i}\right)\pi_{\tilde{B}L}\left(x_{i}\right)+\pi_{\tilde{A}U}\left(x_{i}\right)\pi_{\tilde{B}U}\left(x_{i}\right)}{\sqrt{\mu_{\tilde{A}L}^{2}\left(x_{i}\right)+\mu_{\tilde{A}U}^{2}\left(x_{i}\right)+\nu_{\tilde{A}L}^{2}\left(x_{i}\right)+\nu_{\tilde{A}U}^{2}\left(x_{i}\right)+\pi_{\tilde{A}L}^{2}\left(x_{i}\right)+\pi_{\tilde{A}U}^{2}\left(x_{i}\right)}\cdot \left|H\right|}, \end{array}$$

where

(7) $$\begin{array}{c} \left|H\right|=\sqrt{\mu_{\tilde{B}L}^{2}\left(x_{i}\right)+\mu_{\tilde{B}U}^{2}\left(x_{i}\right)+\nu_{\tilde{B}L}^{2}\left(x_{i}\right)+\nu_{\tilde{B}U}^{2}\left(x_{i}\right)+\pi_{\tilde{B}L}^{2}\left(x_{i}\right)+\pi_{\tilde{B}U}^{2}\left(x_{i}\right)}. \end{array}$$

The cosine similarity measure between two IVIFs $\tilde{A}$ and $\tilde{B}$ satisfies the following properties:

1. $0\leq C_{\text{IVIFS}}\left(\tilde{A},\tilde{B}\right)\leq 1$
2. $C_{\text{IVIFS}}\left(\tilde{A},\tilde{B}\right)=C_{\text{IVIFS}}\left(\tilde{B},\tilde{A}\right)$
3. $C_{\text{IVIFS}}\left(\tilde{A},\tilde{B}\right)=1\text{ if}\tilde{A}=\tilde{B}$

## 3. Cosine Similarity Measure with Hybrid Intuitionistic Fuzzy Information

In this section, we will propose the cosine similarity measure with hybrid intuitionistic fuzzy information (*C*_HIFS_) and some properties are also discussed.

*Definition 5*. Let *A* be fuzzy set (FS) in *X*={*x*_1_, *x*_2_, ..., *x*_*n*_}, I and II be two subsets of the attribute set *X*, such that I ∪ II=*X*, I∩II=*ϕ*. If *x*_*j*_ ∈ I, the value of fuzzy set *A* is characterized by IFSs, if *x*_*j*_ ∈ II, the values of fuzzy set *A* is characterized by IVIFSs, then *A* is called hybrid intuitionistic fuzzy sets (HIFSs).

*Definition 6*. Let *A*={(*x*_*j*_, *μ*_*A*_(*x*_*j*_), *ν*_*A*_(*x*_*j*_))|*x*_*j*_ ∈ *X*} and *B*={(*x*_*j*_, *μ*_*B*_(*x*_*j*_), *ν*_*B*_(*x*_*j*_))|*x*_*j*_ ∈ *X*} be two hybrid intuitionistic fuzzy sets, such that if the same attributes *x*_*j*_ ∈ I, (*μ*_*A*_(*x*_*j*_), *ν*_*A*_(*x*_*j*_)), and (*μ*_*B*_(*x*_*j*_), *ν*_*B*_(*x*_*j*_)) are IFSs, if the same attribute *x*_*j*_ ∈ II, ([*μ*_*AL*_(*x*_*j*_), *μ*_*AU*_(*x*_*j*_)], [*ν*_*AL*_(*x*_*j*_), *ν*_*AU*_(*x*_*j*_)]), and ([*μ*_*BL*_(*x*_*j*_), *μ*_*BU*_(*x*_*j*_)], [*ν*_*BL*_(*x*_*j*_), *ν*_*BU*_(*x*_*j*_)]) are IVIFSs, which we call *A* and *B* the same type hybrid intuitionistic fuzzy sets.

*Definition 7*. Suppose *A* and *B* are the same type hybrid intuitionistic fuzzy sets, that is, if *x*_*j*_ ∈ I, (*μ*_*A*_(*x*_*j*_), *ν*_*A*_(*x*_*j*_)), and (*μ*_*B*_(*x*_*j*_), *ν*_*B*_(*x*_*j*_)) are IFSs, if the same attribute *x*_*j*_ ∈ II, ([*μ*_*AL*_(*x*_*j*_), *μ*_*AU*_(*x*_*j*_)], [*ν*_*AL*_(*x*_*j*_), *ν*_*AU*_(*x*_*j*_)]), and ([*μ*_*BL*_(*x*_*j*_), *μ*_*BU*_(*x*_*j*_)], [*ν*_*BL*_(*x*_*j*_), *ν*_*BU*_(*x*_*j*_)]) are IVIFSs, then the cosine similarity measure between hybrid intuitionistic fuzzy sets *A* and *B* is defined as follows:

(8) $$\begin{array}{c} C_{\text{HIFS}}\left(A,B\right)=\frac{1}{n}\left[\underset{x_{i}\in \text{I}}{\sum }\frac{\mu_{A}\left(x_{i}\right)\mu_{B}\left(x_{i}\right)+\nu_{A}\left(x_{i}\right)\nu_{B}\left(x_{i}\right)+\pi_{A}\left(x_{i}\right)\pi_{B}\left(x_{i}\right)}{\sqrt{\mu_{A}^{2}\left(x_{i}\right)+\nu_{A}^{2}\left(x_{i}\right)+\pi_{A}^{2}\left(x_{i}\right)}\sqrt{\mu_{B}^{2}\left(x_{i}\right)+\nu_{B}^{2}\left(x_{i}\right)+\pi_{B}^{2}\left(x_{i}\right)}}+\underset{x_{i}\in \text{II}}{\sum }\frac{\mu_{AL}\left(x_{i}\right)\mu_{BL}\left(x_{i}\right)+\mu_{AU}\left(x_{i}\right)\mu_{BU}\left(x_{i}\right)+\nu_{AL}\left(x_{i}\right)\nu_{BL}\left(x_{i}\right)+\nu_{AU}\left(x_{i}\right)\nu_{BU}\left(x_{i}\right)+\pi_{AL}\left(x_{i}\right)\pi_{BL}\left(x_{i}\right)+\pi_{AU}\left(x_{i}\right)\pi_{BU}\left(x_{i}\right)}{\sqrt{\mu_{AL}^{2}\left(x_{i}\right)+\mu_{AU}^{2}\left(x_{i}\right)+\nu_{AL}^{2}\left(x_{i}\right)+\nu_{AU}^{2}\left(x_{i}\right)+\pi_{AL}^{2}\left(x_{i}\right)+\pi_{AU}^{2}\left(x_{i}\right)}\cdot \sqrt{\mu_{BL}^{2}\left(x_{i}\right)+\mu_{BU}^{2}\left(x_{i}\right)+\nu_{BL}^{2}\left(x_{i}\right)+\nu_{BU}^{2}\left(x_{i}\right)+\pi_{BL}^{2}\left(x_{i}\right)+\pi_{BU}^{2}\left(x_{i}\right)}}\right.. \end{array}$$

***Remark 2*.**

If I=*ϕ*, then *C*_HIFS_ measure is reduced to *C*_IVIFS_ measure.

***Remark 3*.**

If II=*ϕ*, then *C*_HIFS_ measure is reduced to *C*_IFS_ measure.

**Theorem 1 .**

*The cosine similarity measure between two hybrid intuitionistic fuzzy setsAandBsatisfies the following properties*:

1. 0 ≤ *C*_HIFS_(*A*, *B*) ≤ 1
2. *C*_HIFS_(*A*, *B*)=*C*_HIFS_(*B*, *A*)
3. *C*_HIFS_(*A*, *B*)=1 if *A*=*B*

*Proof*

1. It is obvious that the property (1) is true according to the cosine value in [0,1]
2. Because the multiplication of numbers satisfies the commutative law, if the positions of *A* and *B* are exchanged in the computation of cosine measure, the result values will not change, so the property (3) is true.
3. If *A*=*B*, *x*_*i*_ ∈ I, we have *μ*_*A*_(*x*_*i*_)=*μ*_*B*_(*x*_*i*_) and *ν*_*A*_(*x*_*i*_)=*ν*_*B*_(*x*_*i*_).

If *A*=*B*, *x*_*i*_ ∈ II, we have *μ*_*AL*_(*x*_*i*_)=*μ*_*BL*_(*x*_*i*_), *μ*_*AU*_(*x*_*i*_)=*μ*_*BU*_(*x*_*i*_), *ν*_*AL*_(*x*_*i*_)=*ν*_*BL*_(*x*_*i*_), and *ν*_*AU*_(*x*_*i*_)=*ν*_*BU*_(*x*_*i*_), then *C*_HIFS_(*A*, *B*)=1 is obvious obtained.

## 4. Multiple-Attribute Group Decision-Making with the Cosine Similarity Measure between Hybrid Intuitionstic Fuzzy Sets

In this section, we will apply the *C*_HIFS_ measure between hybrid intuitionistic fuzzy sets to medical diagnosis. The *C*_HIFS_ measure can be applied in many situations, such as pattern recognition, medical diagnosis, and so on. The main motivation for considering this model is that the representation of the decision information is very complex. We need several doctors correctly to evaluate the symptoms of the disease. The doctor usually provides his/her preferences for symptoms with IFSs or IVIFSs. Suppose that doctors are good at different diagnostic skills, we can obtain the weights of doctors based on the projection of individual decision on the ideal decision; then, all individual diagnosis decisions are aggregated into a collective one. At last, we apply the *C*_HIFS_ measure to medical diagnosis.

In a given pathology, suppose that a set of symptoms *S*=(*s*_1_, *s*_2_,…, *s*_*n*_), a set of diagnoses *A*=(*A*_1_, *A*_2_,…, *A*_*m*_) and a set of medical experts *E*=(*e*_1_, *e*_2_,…, *e*_*t*_). Assume that a patient has all the symptoms, which can be represented by the hybrid intuition fuzzy set $\tilde{B}$, our aim is to diagnose what kind of diagnoses the patient $\tilde{B}$ belong to.

In order to solve this problem, we first introduce some relevant concepts.

*Definition 8*. Let *A*′=(*a*_*ij*_)_*m*×*n*_=(*μ*_*A*_*i*__(*x*_*j*_), *ν*_*A*_*i*__(*x*_*j*_))_*m*×*n*_ be a decision matrix, I and II be two subsets of the attribute set *X*={*x*_*j*_|*j*=1,2,…, *n*), such that I ∪ II=*X* and I∩II=∅. If the attribute *x*_*j*_ ∈ *Ι*, then the evaluation values *a*_*ij*_ are IFSs, if the attribute *x*_*j*_ ∈ II, then the evaluation values *a*_*ij*_ are IVIFSs. In this case, *A*′ is called a hybrid intuitionistic fuzzy matrix.

*Definition 9*. Let *A*′=(*A*_1_, *A*_2_,…,*A*_*m*_)^*T*^ and *B*′=(*B*_1_, *B*_2_,…,*B*_*m*_)^*T*^ be two hybrid intuitionistic fuzzy matrices, where *A*_*i*_=(*a*_*i*1_, *a*_*i*2_,…, *a*_*in*_) and *B*_*i*_=(*b*_*i*1_, *b*_*i*2_,…, *b*_*in*_)(*i*=1,2,…*m*), if they satisfy the following conditions:

1. *a*_*ij*_=(*μ*_*A*_*i*__(*x*_*j*_), *ν*_*A*_*i*__(*x*_*j*_)) about *x*_*j*_ in *A*_*i*_ if and only if *b*_*ij*_=(*μ*_*Bi*_(*x*_*j*_), *ν*_*Bi*_(*x*_*j*_)) about *x*_*j*_ in *B*_*i*_
2. *a*_*i*_=([*μ*_*A*_*i*_*L*_(*x*_*j*_), *μ*_*A*_*i*_*U*_(*x*_*j*_)], [*ν*_*A*_*i*_*L*_(*x*_*j*_), *ν*_*A*_*i*_*U*_(*x*_*j*_)]) about *x*_*j*_ in *A*_*i*_ if and only if *b*_*ij*_=([*μ*_*B*_*i*_*L*_(*x*_*j*_), *μ*_*B*_*i*_*U*_(*x*_*j*_)], [*ν*_*B*_*i*_*L*_(*x*_*j*_), *ν*_*B*_*i*_*U*_(*x*_*j*_)]) about *x*_*j*_ in *B*_*i*_, then *A*_*i*_ and *B*_*i*_ are the same type vector, *A*′ and *B*′ are the same type hybrid intuitionistic fuzzy matrices.

Now, suppose that a set of diagnoses *A*′=(*A*_1_, *A*_2_,…, *A*_*m*_), where *A*_*i*_ is represented by IFS *A*_*i*_=(*x*_*j*,_ *μ*_*A*_*i*__(*x*_*j*_), *ν*_*A*_*i*__(*x*_*j*_)) or IVIFS *A*_*i*_=(*x*_*j*,_[*μ*_*A*_*i*_*L*_(*x*_*j*_), *μ*_*A*_*i*_*U*_(*x*_*j*_)], [*ν*_*A*_*i*_*L*_(*x*_*j*_), *ν*_*A*_*i*_*U*_(*x*_*j*_)])(*i*=1,2,…, *m*). We should diagnose what kind of disease the patient $\tilde{B}$ belongs to. Furthermore, assume that the patient $\tilde{B}$ is represented by the same type intuitionistic fuzzy set as *A*_*i*_. In the following, we will present the method for application of *C*_HIFS_ measure to medical diagnosis, which involves the following steps:

*Step 1*. Each medical expert provides his/her individual decision matrix about the relation between the diagnosis and the symptoms.

*Step 2*. According to the expert's diagnostic decision matrix *R*_*k*_=(*r*_*ij*_^*k*^)_*m*×*n*_, the ideal decision information should be close to the opinions of most doctors; then, we define the ideal relation *R* ^*∗*^=(*r*_*ij*_^*∗*^)_*m*×*n*_ between the diagnosis *A*_*i*_(*i*=1,2,…, *m*) and the symptom *s*_*j*_(*j*=1,2,…, *n*) as follows:

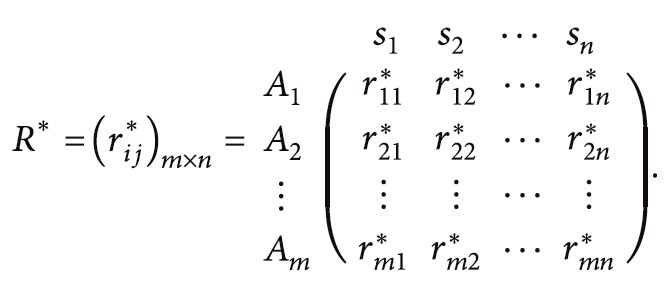
(9)

(10) $$\begin{array}{c} \text{If}j\in Ι,r_{ij}^{*}=\left(\mu_{ij}^{*},\nu_{ij}^{*}\right)=\left(\frac{1}{t}\overset{t}{\underset{k=1}{\sum }}\mu_{ij}^{\left(k\right)},\frac{1}{t}\overset{t}{\underset{k=1}{\sum }}\nu_{ij}^{\left(k\right)}\right). \end{array}$$

(11) $$\begin{array}{c} \text{If}j\in II,r_{ij}^{*}=\left({\tilde{\mu }}_{ij}^{*},{\tilde{\nu }}_{ij}^{*}\right)=\left(\left[\frac{1}{t}\overset{t}{\underset{k=1}{\sum }}\mu_{ijL}^{\left(k\right)},\frac{1}{t}\overset{t}{\underset{k=1}{\sum }}\mu_{ijU}^{\left(k\right)}\right],\left[\frac{1}{t}\overset{t}{\underset{k=1}{\sum }}\nu_{ijL}^{\left(k\right)},\frac{1}{t}\overset{t}{\underset{k=1}{\sum }}\nu_{ijU}^{\left(k\right)}\right]\right). \end{array}$$

*Step 3*. Medical experts may give unreasonable assessments when they encounter unfamiliar symptoms. So, it is not very reasonable to assume that each expert has equal weights. In order to obtain a reasonable evaluation, the weights of medical experts under different attributes are obtained by the projection of the individual evaluation on the ideal evaluation *r*_*ij*_^*∗*^. The greater the weight of the expert is, the closer the evaluation value is to the ideal evaluation. The projection of each decision on the ideal decision is given by

(12) $$\begin{array}{c} \text{if}j\in Ι,\mathrm{Pr}j_{r_{ij}^{*}}^{r_{ij}^{\left(k\right)}}=\frac{\mu_{ij}^{\left(k\right)}\mu_{ij}^{*}+\nu_{ij}^{\left(k\right)}\nu_{ij}^{*}+\pi_{ij}^{\left(k\right)}\pi_{ij}^{*}}{\sqrt{\mu_{ij}^{*2}+\nu_{ij}^{*2}+\pi_{ij}^{*2}}}, \end{array}$$

(13) $$\begin{array}{c} \text{if}j\in \text{II,}\mathrm{Pr}j_{r_{ij}^{*}}^{r_{ij}^{\left(k\right)}}=\frac{\mu_{ijL}^{\left(k\right)}\mu_{ijL}^{*}+\mu_{ijU}^{\left(k\right)}\mu_{ijU}^{*}+\nu_{ijL}^{\left(k\right)}\nu_{ijL}^{*}+\nu_{ijU}^{\left(k\right)}\nu_{ijU}^{*}+\pi_{ijL}^{\left(k\right)}\pi_{ijL}^{*}+\pi_{ijU}^{\left(k\right)}\pi_{ijU}^{*}}{\sqrt{\mu_{ijL}^{*2}+\mu_{ijU}^{*2}+\nu_{ijL}^{*2}+\nu_{ijU}^{*2}+\pi_{ijL}^{*2}+\pi_{ijU}^{*2}}}. \end{array}$$

Then the weight of medical expert's evaluation on different symptoms can be defined as

(14) $$\begin{array}{c} w_{ij}^{\left(k\right)}=\frac{\mathrm{Pr}j_{r_{ij}^{*}}r_{ij}^{\left(k\right)}}{\sum_{k=1}^{t}\mathrm{Pr}j_{r_{ij}^{*}}r_{ij}^{\left(k\right)}},\;k=1,2,\ldots ,t;i=1,2,\ldots ,m;j=1,2,\ldots ,n. \end{array}$$

*Step 4*. According to the recognition principle of maximum degree of cosine similarity measure, the process of diagnosis $\tilde{B}$ to *A*_*k*_ is derived by $k=\underset{1\leq i\leq m}{\text{Max}}\left(C_{\text{HIFS}}\left(A_{i},\tilde{B}\right)\right)$.

## 5. Numerical Example

In this section, the proposed cosine similarity measure between hybrid IFSs is applied in medical diagnosis to demonstrate its effectiveness.

### 5.1. Illustration of the Cosine Similarity Measures for Hybrid IFSs

Assume that a set of diagnosis *A*={*A*_1_(viral  fever), *A*_2_(typhoid), *A*_3_(stomach  problem), *A*_4_(chest  problem)} and a set of symptoms *S*={*s*_1_(temperature), *s*_2_(stomach  pain), *s*_3_(cough), *s*_4_(chest  pain)}. Suppose a patient has all the symptoms, which can be represented by the following hybrid intuitionistic fuzzy information (data obtained through a survey of doctors):

(15) $$\begin{array}{c} \tilde{B}=\left\{\left(s_{1},0.5,0.4\right),\left(s_{2},0.6,0.2\right),\left(s_{3},\left[0.5,0.6\right],\left[0.2,0.3\right]\right),\left(s_{4},0.4,0.2\right)\right\}. \end{array}$$

There are three medical experts evaluate each diagnosis with all the symptoms, which are represented by the hybrid IFSs, the results are shown in Tables 1 [Table tab2]–3.

By step 3 in Section 4, applying (12)–(14), we can calculate the weights of each medical expert for the diagnosis with respect to different symptoms, which are obtained in Tables 5 [Table tab6]–7.

From the previous formula *C*_HIFS_(*A*′, *B*), we can calculate the cosine similarity measure between ${\tilde{A}}_{i}\left(i=1,2,3,4\right)$ and $\tilde{B}$ as follows:

(16) $$\begin{array}{c} C_{\text{HIFS}}\left({\tilde{A}}_{1},\tilde{B}\right)=0.9674,C_{HIFS}\left({\tilde{A}}_{2},\tilde{B}\right)=0.9477,\\ C_{\text{HIFS}}\left({\tilde{A}}_{3},\tilde{B}\right)=0.9140,C_{HIFS}\left({\tilde{A}}_{4},\tilde{B}\right)=0.9511. \end{array}$$

We can conclude that the diagnosis of the patient $\tilde{B}$ is viral fever (*A*_1_).

### 5.2. Comparison Analysis

In this subsection, the existing cosine similarity measure is used to compare with the same numerical example. In the numerical example, the decision information is represented with hybrid IFS, we can transform it into a unified form. For example, the relation between the diagnosis and the symptoms under the attribute *s*_3_ of experts is IVIFSs, and if we use the cosine similarity measure *C*_IFS_ proposed by Ye [7] to calculate the numerical example, we should convert the corresponding IVIFSs to IFS according to the midpoints of IVIFSs. For example, ([0.4, 0.6], [0.1, 0.3]) can be converted to (0.5). Then using the cosine similarity measure *C*_IFS_ proposed by Ye [7], we can obtain the corresponding cosine similarity measure values: $C_{\text{IFS}}\left(A_{1},\tilde{B}\right)=0.9691$, $C_{\text{IFS}}\left(A_{2},\tilde{B}\right)=0.9546$, $C_{\text{IFS}}\left(A_{3},\tilde{B}\right)=0.9377$, and $C_{IFS}\left(A_{4},\tilde{B}\right)=0.9586$. That is to say, the diagnosis of the patient $\tilde{B}$ is still the viral fever *A*_1_. The proposed cosine similarity between hybrid IFS in this paper produces the same results as the existing methods. This means that the proposed method is feasible and effective, and it has some advantages in solving multiple criteria decision-making problems. On one hand, the method is more convenient to make decision for decision makers, who can express their preferences over the decision information by IFS or IVIFS simultaneously. On the other hand, because the information conversion will be lost in decision-making process, there are no information conversions between IFSs and IVIFSs in this model, the alternatives will be ranked directly based on the original decision information.

## 6. Conclusion

The paper proposed the cosine similarity measure between hybrid intuitionistic fuzzy sets, and the proposed method would be quite good for some real-world applications, such as pattern recognition and medical diagnosis. Through the proposed cosine similarity measure, we can classify the patient $\tilde{B}$ in one of the diagnosis *A*_1_, *A*_2_,…*A*_*m*_. Finally, a numerical example illustrated the application and efficiency of the developed approach, which is also compared to the existing methods. In future research, we expect to develop further extensions of the *C*_HIFS_ measure by adding the new characteristic, such as ordered weighted averaging operator, and we will also consider other applications of the proposed *C*_HIFS_ measure.

## Figures and Tables

**Table 1:** The relation between the diagnosis and the symptoms—expert 1.

	*s*_1_	*s*_2_	*s*_3_	*s*_4_
*A*_1_	(0.5, 0.4)	(0.5, 0.3)	([0.4, 0.6], [0.1, 0.3])	(0.4, 0.4)
*A*_2_	(0.7, 0.3)	(0.7, 0.2)	([0.3, 0.5], [0.4, 0.5])	(0.6, 0.2)
*A*_3_	(0.8, 0.1)	(0.6, 0.4)	([0.6, 0.7], [0.2, 0.3])	(0.6, 0.3)
*A*_4_	(0.7, 0.2)	(0.5, 0.2)	([0.5, 0.7], [0.1, 0.2])	(0.5, 0.3)

**Table 2 tab2:** The relation between the diagnosis and the symptoms—expert 2.

	*s*_1_	*s*_2_	*s*_3_	*s*_4_
*A*_1_	(0.4, 0.5)	(0.6, 0.2)	([0.5, 0.6], [0.2, 0.3])	(0.3, 0.4)
*A*_2_	(0.5, 0.2)	(0.7, 0.2)	([0.4, 0.7], [0.1, 0.3])	(0.7, 0.1)
*A*_3_	(0.6, 0.2)	(0.5, 0.1)	([0.5, 0.7], [0.1, 0.2])	(0.6, 0.2)
*A*_4_	(0.7, 0.1)	(0.4, 0.3)	([0.3, 0.6], [0.2, 0.4])	(0.4, 0.3)

**Table 3:** The relation between the diagnosis and the symptoms—expert 3.

	*s*_1_	*s*_2_	*s*_3_	*s*_4_
*A*_1_	(0.5, 0.3)	(0.6, 0.2)	([0.4, 0.6], [0.2, 0.3])	(0.5, 0.4)
*A*_2_	(0.7, 0.2)	(0.4, 0.4)	([0.5, 0.7], [0.1, 0.3])	(0.6, 0.3)
*A*_3_	(0.6, 0.3)	(0.7, 0.3)	([0.6, 0.8], [0.1, 0.2])	(0.7, 0.2)
*A*_4_	(0.5, 0.2)	(0.5, 0.3)	([0.3, 0.6], [0.1, 0.4])	(0.6, 0.1)

According to step 2 in Section 4, applying (10) and (11), respectively, the ideal relation between the diagnosis and the symptoms are shown in Table 4.

**Table 4:** The ideal relation between the diagnosis and the symptoms.

	*s*_1_	*s*_2_	*s*_3_	*s*_4_
*A*_1_^*∗*^	(0.467, 0.4)	(0.567, 0.233)	([0.433, 0.6], [0.167, 0.3])	(0.4, 0.4)
*A*_2_^*∗*^	(0.633, 0.233)	(0.6, 0.267)	([0.4, 0.633], [0.2, 0.367])	(0.633, 0.2)
*A*_3_^*∗*^	(0.667, 0.2)	(0.6, 0.267)	([0.567, 0.733], [0.133, 0.233])	(0.633, 0.233)
*A*_4_^*∗*^	(0.633, 0.167)	(0.467, 0.267)	([0.367, 0.633], [0.133, 0.333])	(0.5, 0.233)

**Table 5:** The weights of the medical expert 1 for *A*_*i*_ with respect to *s*_*j*_.

	*s*_1_	*s*_2_	*s*_3_	*s*_4_
*A*_1_	0.3427	0.3155	0.3391	0.3333
*A*_2_	0.3614	0.3614	0.3052	0.3223
*A*_3_	0.3761	0.3466	0.3293	0.3263
*A*_4_	0.3458	0.3395	0.3314	0.3313

**Table 6 tab6:** The weights of the medical expert 2 for *A*_*i*_ with respect to *s*_*j*_.

	*s*_1_	*s*_2_	*s*_3_	*s*_4_
*A*_1_	0.3371	0.3423	0.3311	0.3148
*A*_2_	0.2841	0.3614	0.3474	0.3531
*A*_3_	0.3097	0.2821	0.3196	0.3193
*A*_4_	0.3594	0.3210	0.3276	0.3106

**Table 7:** The weights of the medical expert 3 for *A*_*i*_ with respect to *s*_*j*_.

	*s*_1_	*s*_2_	*s*_3_	*s*_4_
*A*_1_	0.3202	0.3422	0.3298	0.3519
*A*_2_	0.3545	0.2772	0.3474	0.3246
*A*_3_	0.3142	0.3713	0.3511	0.3544
*A*_4_	0.2948	0.3395	0.3410	0.3581

When the weight values of the experts are determined, the aggregated evaluating decision results provided by different experts are obtained in Table 8.

**Table 8:** The aggregated relation between the diagnosis and the symptom.

	*s*_1_	*s*_2_	*s*_3_	*s*_4_
${\tilde{A}}_{1}$	(0.4683, 0.3933)	(0.5708, 0.2273)	([0.4351, 0.6], [0.1581, 0.3])	(0.4093, 0.4)
${\tilde{A}}_{2}$	(0.5316, 0.2316)	(0.6364, 0.2424)	([0.4097, 0.6494], [0.1527, 0.3506])	(0.6386, 0.1786)
${\tilde{A}}_{3}$	(0.6918, 0.175)	(0.6172, 0.2431)	([0.5704, 0.7398], [0.1256, 0.2286])	(0.6388, 0.2283)
${\tilde{A}}_{4}$	(0.6513, 0.1559)	(0.4699, 0.2614)	([0.3739, 0.6364], [0.1255, 0.3179])	(0.5115, 0.2024)

## Data Availability

No data were used to support this study.

## Abbreviations

IFS: Intuitionistic fuzzy set
IVIFS: Interval-valued intuitionistic fuzzy set
HIFS: Hybrid intuitionistic fuzzy set
*C*_IFS_: Cosine similarity measure for intuitionistic fuzzy set
*C*_IVIFS_: Cosine similarity measure for interval-valued intuitionistic fuzzy set
*C*_HIFS_: Cosine similarity measure for hybrid intuitionistic fuzzy set.
